# The amphibians and reptiles of Colima, Mexico, with a summary of their conservation status

**DOI:** 10.3897/zookeys.927.50064

**Published:** 2020-04-16

**Authors:** Julio A. Lemos-Espinal, Geoffrey R. Smith, Leland J.S. Pierce, Charles W. Painter

**Affiliations:** 1 Laboratorio de Ecología UBIPRO, FES Iztacala UNAM, Avenida Los Barrios 1, Los Reyes Iztacala, Tlalnepantla Estado de México, 54090, Mexico Universidad Nacional Autónoma de México Tlalnepantla Mexico; 2 Department of Biology, Denison University, Granville, Ohio, USA Denison University Granville United States of America; 3 New Mexico Department of Game and Fish, P.O. Box 25112, Santa Fe, New Mexico 87504, USA New Mexico Department of Game and Fish Sante Fe United States of America

**Keywords:** checklist, crocodilians, frogs, herpetofauna, lizards, salamanders, snakes, turtles

## Abstract

Colima is the fourth smallest Mexican state, covering only 0.3% of the surface area of Mexico, but due to the remarkable diversity of physiographic and environmental conditions present in Colima it contains a high biological diversity. We generated an up-to-date herpetofaunal checklist for Colima, with a summary of the conservation status of Colima’s amphibians and reptiles. Our checklist contains a total of 153 species of amphibians and reptiles (three introduced). Thirty-nine are amphibians and 114 are reptiles. More than half of Colima’s herpetofauna are Mexican endemics (66.7% of amphibians, 67.5% of reptiles). Less than 25% of the amphibian and reptile species in Colima are in protected categories according to the IUCN Red List and SEMARNAT. The reptiles in the Marine and Revillagigedo Archipelago regions are the most threatened taxa of the Colima herpetofauna. Colima shares > 80% of its herpetofauna with its neighboring states, Jalisco and Michoacán.

## Introduction

A number of Mexican states still lack comprehensive species lists of amphibians and reptiles. One such state is Colima, which despite being the fourth smallest Mexican state, covering only 0.3% of the surface area of Mexico, has, as reported here, a rich herpetofauna represented by 150 native species (38 amphibians and 112 reptiles), in part due to the remarkable diversity of physiographic and environmental conditions present in Colima.

The interest in the study of amphibians and reptiles of the state of Colima dates from 1700, the year in which the first official record of a herpetological specimen collected in Colima (*Rana pustulosa* – MVZ-A20941). More than 200 years later, Oliver (1937) reported 61 species from Colima. Duellman (1958) subsequently listed 82 amphibian and reptile species from the lowlands of Colima, and Painter (1976) studied the distribution of amphibians and reptiles in Colima. More recently, Reyes-Velasco et al. (2009) reported new state records for 21 species of amphibians and reptiles from Colima. In addition, there have been several new species recently described or elevated to species status from Colima. Bryson et al. (2014) described *Crotalus campbelli* from the Sierra de Manantlán of southwestern Jalisco and northern Colima and elevated *Crotalus triseriatus armstrongi* to a full species status (*C. armstrongi*). Reyes-Velasco et al. (2015) described *Eleutherodactylus grunwaldi* from the state of Colima. Grünwald et al. (2018) described two new species of *Eleutherodactylus* from Colima, *E. colimotl* and *E. manantlanensis*, the last one endemic to Colima.

Given these recent additions and changes in the known species of amphibians and reptiles of Colima, we have conducted a comprehensive review of the specimens and documented species of amphibians and reptiles from Colima to provide an up-to-date herpetofaunal checklist from Colima. In addition, we review and summarize the conservation status of these amphibians and reptiles as a potential guide to future conservation and management efforts focused on the amphibians and reptiles of Colima.

### Physiography of Colima

**Figure 1.**
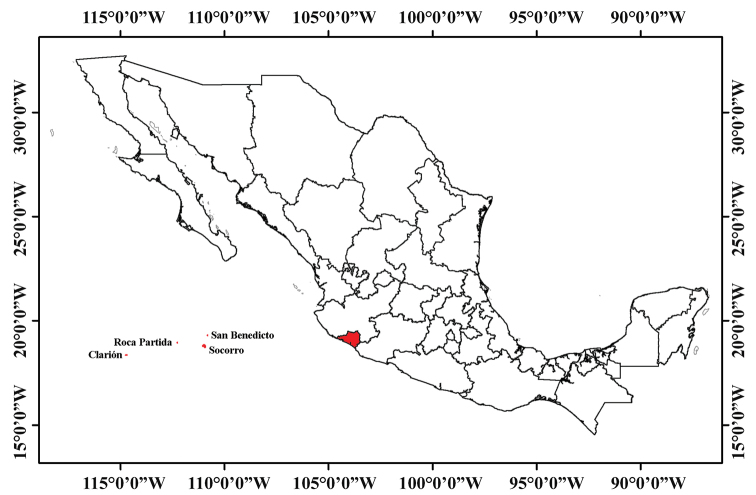
Map of Mexico with the state of Colima shown in red (modified from INEGI 2018).

Colima is one of the smallest states in Mexico, covering 5,627 km^2^ between 19°30'45"N and 18°41'03"N, and -103°29'11"W and -104°41'26"W. Colima is located in central-western Mexico, in the middle of the Pacific Coast of Mexico (Fig. 1). Colima is bordered by Jalisco to the north and east, Michoacán to the southeast, and the Pacific Ocean to the west and south. The Revillagigedo Archipelago is part of the state of Colima and includes the islands of Socorro, San Benedicto, Clarion, and Roca Partida (INEGI 2017), lies approximately 390 km southwest of Cabo San Lucas, the southern tip of the Baja California Peninsula, and 720 to 970 km west of Manzanillo, northwestern Colima (https://en.wikipedia.org/wiki/Revillagigedo_Islands – accessed 10 October 2019).

**Figure 2.**
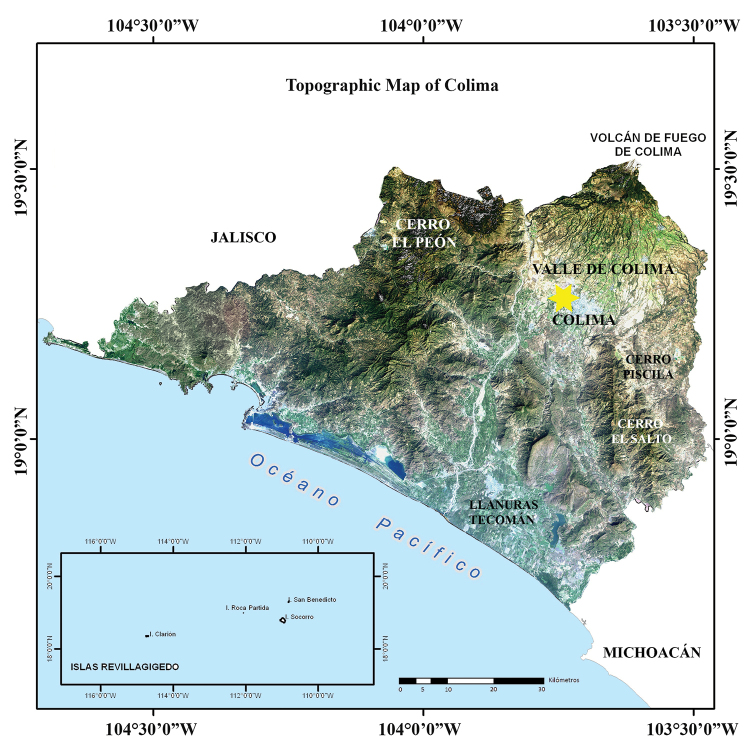
Satellite map showing the topographic features of Colima (from Comisión Nacional para el Conocimiento y Uso de la Biodiversidad 2008).

**Figure 3.**
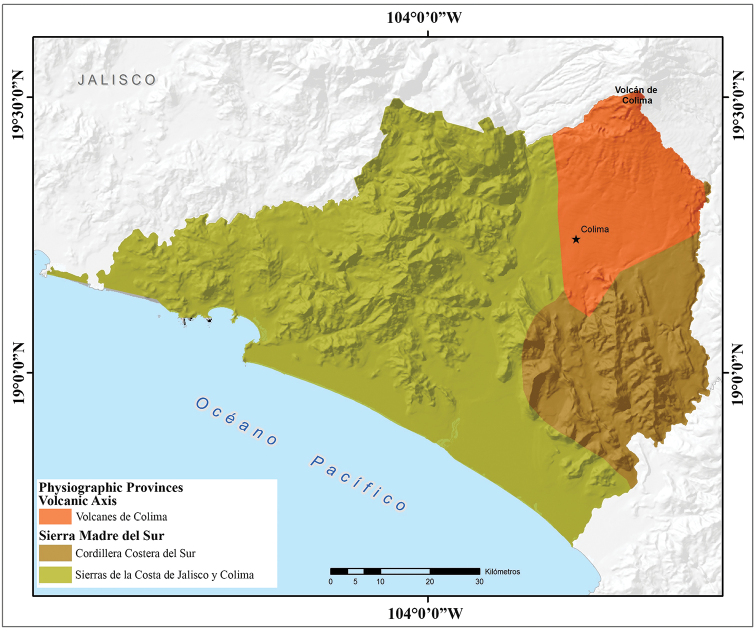
Physiographic provinces of the state of Colima, Mexico (modified from Cervantes-Zamora et al. 1990).

Almost three quarters of the surface area of Colima is covered by mountains and hills, producing a heterogeneous topography in the state (Fig. 2). This complex topography is represented by two physiographic provinces that are included within the geographic limits of the state: the Volcanic Axis, represented in the state by the subprovince of Volcanes de Colima; and the Sierra Madre del Sur, represented in the state by two subprovinces “Sierras de la Costa de Jalisco y Colima” and “Cordillera Costera del Sur” (Fig. 3). The subprovince of Volcanes de Colima is found in the northern corner of the state in the region known as Valle de Colima, and occupies 16.03% of the state’s surface area. The Colima Volcanos (Nevado de Colima, which actually lies in the state of Jalisco, and Volcán de Colima which lies in the states of Jalisco and Colima) are found in this subprovince. All the northern and northeastern slopes and most of the eastern slopes of these two volcanoes lie in the state of Jalisco. The Valley of Colima, formed from the slopes of the Volcán de Colima, is also found in this region. In Colima the subprovince of Sierras de la Costa de Jalisco and Colima occupies most of the state (62.51% of the surface area). It includes the western mountains, the Marabasco River Basin, the Armeria Valley and the entire Colima coast. In the west-central and southern part of the state that parallels the coast the land is flat, and the Valle de Armeria or Llanuras de Tecomán is found here. Northwestern Colima has mountain ranges intermixed with small valleys. The subprovince of the Cordillera Costera del Sur is also part of the province of the Sierra Madre del Sur and occupies 16.03% of the surface area in extreme eastern Colima. Approximately half of this subprovince is represented by mountain ranges that do not reach 2,000 m elevation, and the other half by valleys, hills, and plains (www.inegi.gob.mx accessed 10 October 2019).

In Colima, the Sierra Madre del Sur consists of four mountain systems. The first system, and the most important, is located in northern Colima and includes Cerro Grande and several hills (Jurípichi or Juluapan, Los Juanillos, La Astilla, El Ocote, El Peón, El Barrigón, San Diego and La Media Luna). The second system runs from the northwestern end of the state southeast to central Colima, and is formed by mountain ranges (the Espinazo del Diablo, El Escorpión , El Tigre, El Aguacate, El Centinela, El Toro and La Vaca) that parallel to the coast between the Marabasco and Armería rivers. The third system is located in central Colima, and consists of hills (Alcomún and Partida, San Miguel and Comala, and San Gabriel or Callejones) that extend to the south between the Armería and Salado rivers. The fourth system is found in southeastern Colima between the Salado and Naranjo or Coahuayana rivers, and includes multiple mountain ranges (Piscila, Volcancillos, La Palmera, El Camichín and Copales) (http://www.inafed.gob.mx/work/enciclopedia/EMM06colima/mediofisico.html – accessed October 10, 2019).

**Figure 4.**
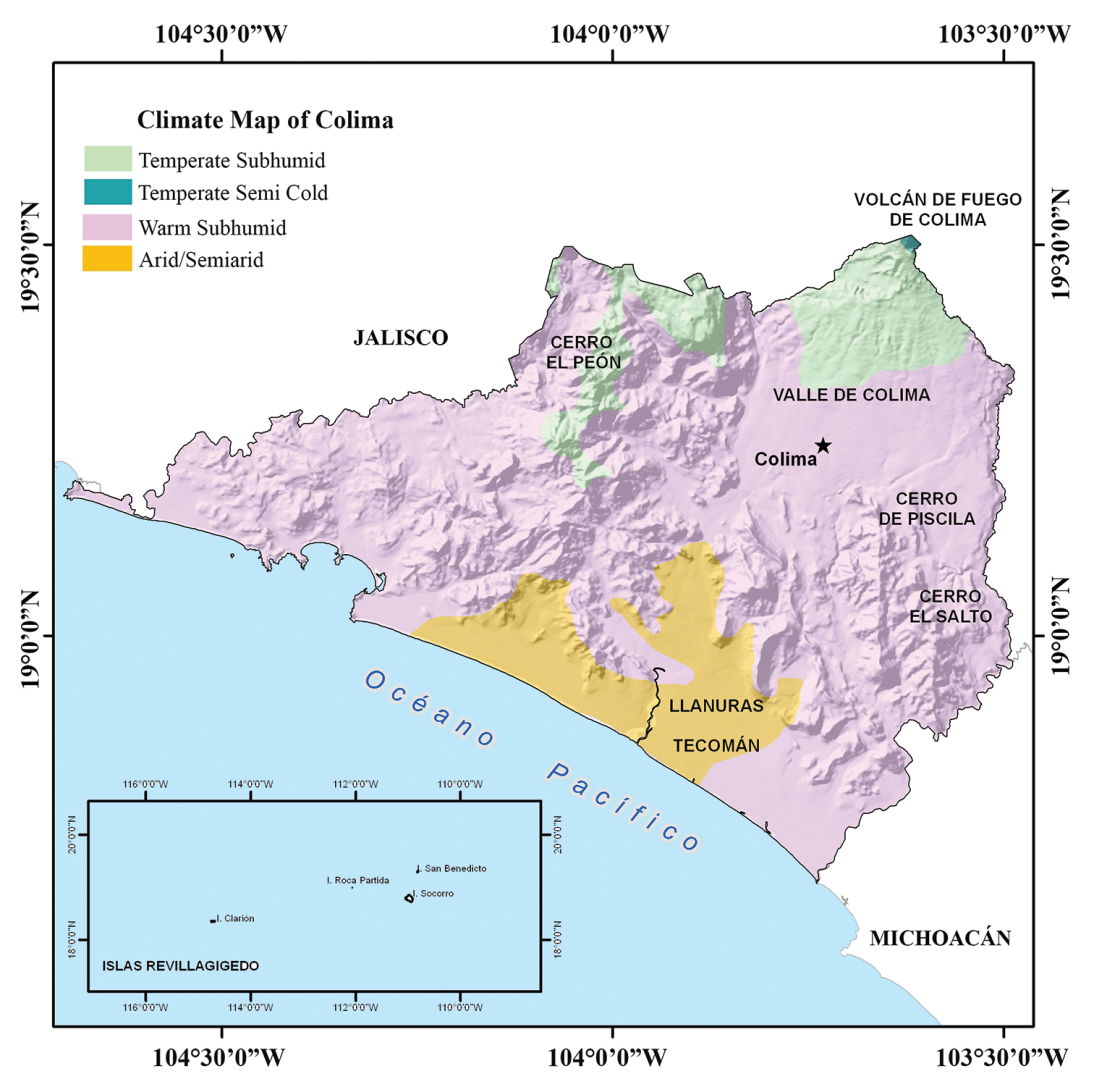
Climate map of the state of Colima, Mexico (modified from García – Comisión Nacional para el Conocimiento y Uso de la Biodiversidad 1998).

The climate of Colima is very diverse (Fig. 4), although relatively high humidity predominates throughout the state. In northern Colima the climate is warm sub-humid, whereas in the mountains there is a sub-humid semi-warm climate and the plains of Tecomán have a semi-dry warm climate. In the coastal area and in the Armería river basin the climate is warm and humid. The average annual temperature ranges around 25 °C, with the maximum of 38 °C and the minimum of 7 °C. Average annual rainfall is 983 mm. Colima’s climate is greatly influenced by the presence of mountains to the west, north and east. The mountain range of Picila creates the southern border of the Valley of Colima, and to the south, the plains of Tecomán end in a low and sandy coast. These mountains, due to their latitude and exposure, allow rainfall to be greater and the climate to differ from the lower elevation parts of the state. In the coastal zone and in the Armería river basin the climate is warm and humid, whereas in the higher elevations in the southern zone it is warm and temperate.

**Figure 5.**
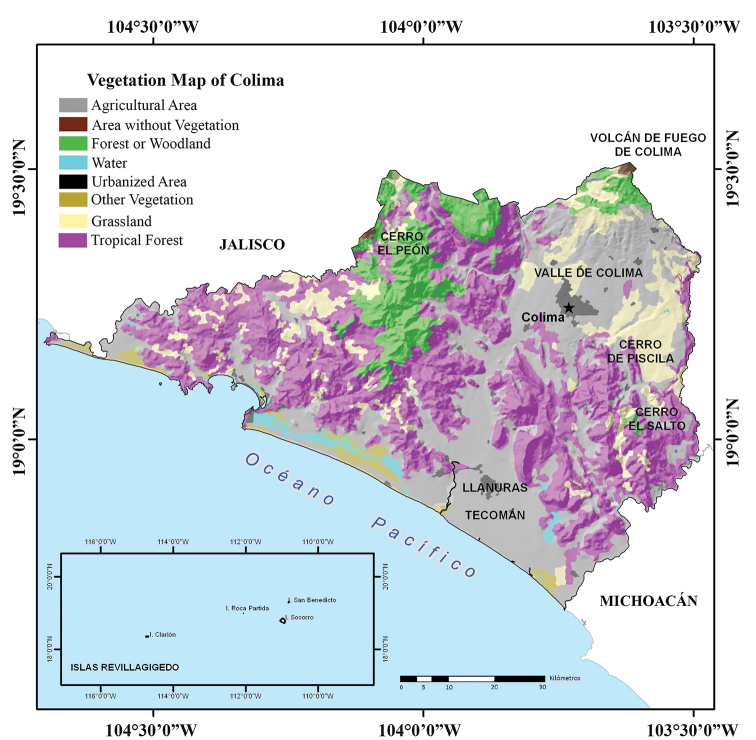
Vegetation map of the state of Colima, Mexico (modified from Dirección General de Geografía – INEGI 2005).

The occurrence of various tropical and temperate floristic elements coupled with variations in the physical environment has resulted in an intricate and complex mosaic of plant associations in Colima (Fig. 5; Schaldach 1963; INEGI 2017). The types of vegetation present in the state are several types of tropical forest, palm groves, savanna, mangrove, coniferous forest, as well as areas of irrigated agriculture. The different types of tropical forest occupy most Colima’s area (74%). Medium subdeciduous forest covers 57% of the state and is present in all municipalities, with the dominant species being highly branched canopy trees, 15 to 25 m high and 50 to 75% of species losing their leaves in the dry season. Medium subperenifolious forest covers 15.4% of Colima. The vegetation is characterized by a height of 20 to 25 m, with 50 to 75% of the species being evergreens. Low deciduous forest covers 1.3% of the area of Colima. The dominant vegetation is low trees from 8 to 12 m high, with abundant leaves that fall in the dry season. Prickly low deciduous forest covers only 1.0% of Colima. It is characterized by the presence of deciduous trees 4 to 8 m tall with thorns. Palmar is only found in the municipalities of Manzanillo and Armería. Mangrove is distributed in the coastal area with coastal lagoons. Savanna is characterized by widely dispersed trees and grasses and is a product of logging or burning of primary communities. Different types of coniferous forests occupy 10.6% of the state’s area.

At the highest elevations in northern Colima, > 50% of the area is forested, so that the agricultural area is limited to 42,700 ha (12,000 ha of irrigation and 30,700 ha of temporary), and due to the topography < 30% can be subjected to mechanized agriculture. The coastal region is characterized as being more favorable for agriculture, including the Tecomán region where the largest area with irrigation infrastructure and plantations with perennial crops is concentrated and mechanized agriculture is used over an area of 92,700 ha (58,400 ha of irrigation and 34,300 ha of temporary).

### Recent taxonomic changes

Acevedo et al. (2016) demonstrated that there were two evolutionary lineages within *Rhinella marina*, one eastern and one western Andean. The eastern populations retained the name *R. marina*, and the western populations were given the revalidated name *R. horribilis*. Duellman et al. (2016) treated two major Hylid clades as genera: *Hyla* which is restricted to the Old World, and *Dryophytes* which is distributed primarily in the New World, including species in Mexico. Reyes-Velasco et al. (2015) described a new species of *Eleutherodactylus* from the Sierra de Manantlán in northern Colima and southwestern Jalisco (*Eleutherodactylus grunwaldi*). In addition, Grünwald et al. (2018) described two new species of *Eleutherodactylus* from Colima, one endemic to the state (*Eleutherodactylus manantlanensis*) and one limited to Colima and western Michoacán (*E. colimotl*). Grünwald et al. (2018) also regarded *E. nivicolimae* as a synonym of *E. rufescens*. Frost et al. (2006) recommended the use of the name *Lithobates* for most New World species of *Rana*, including those in Mexico. However, Yuan et al. (2016) retained all the species of the genera suggested by Frost et al. (2006), including *Lithobates*, in the traditional genus *Rana*, based on clear monophyly of a larger group that includes all of these genera. We therefore follow Yuan et al. (2016) and AmphibiaWeb (2019) in using *Rana* instead of *Lithobates*.

*Plestiodon indubitus* was originally described by Taylor (1933), however Dixon (1969) regarded it as a subspecies of *P. brevirostris*. Feria-Ortiz et al. (2011) subsequently elevated it to full species status, and suggested that the western populations of *P. b. indubitus* from Colima and Jalisco likely represent an undescribed species. We tentatively assign the name *indubitus* to the Colima population until a new name is available. Originally *Holcosus sinister* was described as a subspecies of *H. undulatus* by Smith and Laufe (1946), but it has recently been elevated to full species status by Meza-Lázaro and Nieto-Montes de Oca (2015). Card et al. (2016) resurrected the name *sigma* for the population from María Madre Island, Tres Marías Islands, Nayarit, Mexico described by Smith (1943) as *Constrictor* (= *Boa*) *constrictor sigma*, which was regarded as a junior synonym of *B. c. imperator* by Zweifel (1960). Card et al. (2016) recognized the *Boa* populations from the slopes of the Mexican Pacific as *Boa sigma*, and this is followed here. *Epictia bakewelli* was described as a species by Oliver (1937), and was regarded as a subspecies of *E. goudotti* by Peters et al. (1970). However, Wallach et al. (2014) considered it a full species, and McCranie and Hedges (2016) confirmed its status as a full species. Originally *Rena dugesii* was described as a species by Bocourt (1881), but for a long time it was regarded as a subspecies of *Rena humilis*, however it has recently been regarded as a full species (Wallach et al. 2014). Bryson et al. (2014) described a new species of *Crotalus* from western Jalisco and the Sierra de Manantlán of southwestern Jalisco and northern Colima (*Crotalus campbelli*). They also recognized *C. armstrongi* as a species, which was originally described as a subspecies of *C. triseriatus*.

## Methods

We generated our list of the amphibians and reptiles of Colima using our own field work, a thorough examination of the available literature, checking the amphibian and reptile records for Colima in VertNet.org, and consulting databases from the Comisión Nacional para el Conocimiento y Uso de la Biodiversidad (National Commission for the Understanding and Use of Biodiversity; CONABIO), including records from museum collections listed in Appendix 1.

The amphibian names we use follow Frost (2019) and AmphibiaWeb (2019) (http://amphibiaweb.org) and the reptile names we use follow Uetz and Hošek (2019). We include species in the list only if we could confirm records, either by direct observation or through documented museum records or vouchers. We created species accumulation curves the total herpetofauna, amphibians, and reptiles using the year of the first recorded observation for each species. Species accumulation curves may provide reasonable estimates of the potential species richness of amphibians and reptiles (see Raxworthy et al. 2012). We recorded the conservation status of each species based on the IUCN Red List 2019-2 (IUCN 2019), listing in SEMARNAT (2010), and Environmental Vulnerability Scores (Wilson et al. 2013a,b; Johnson et al. 2015). We determined the number of species shared between Colima and its neighboring states using recent lists of amphibians and reptiles for Jalisco (Cruz-Sáenz et al. 2017) and Michoacán (Alvarado-Díaz et al. 2013).

## Results and discussion

**Figure 6.**
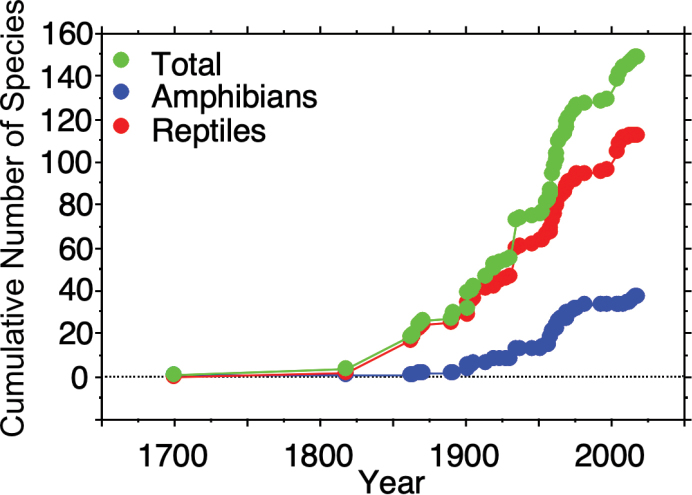
Species accumulation curves for total herpetofauna, amphibians, and reptiles of Colima, Mexico.

A total of 153 species of amphibians and reptiles (three introduced) is found in Colima. Thirty-nine of these species are amphibians (36 anurans [one introduced], two salamanders, and one caecilian), and 114 are reptiles (one crocodilian, 41 lizards [two introduced], 64 snakes, and eight turtles) (Tables 1, 2). These represent 37 families: 12 amphibians (nine anurans, two salamanders, one caecilians), and 25 reptiles (one crocodilian, 12 lizards [one of them introduced], eight snakes, and four turtles); 92 genera: 20 amphibians (17 anurans, two salamanders, one caecilian), and 72 reptiles (one crocodile, 22 lacertilia [two of the introduced], 42 snakes, and six turtles. The introduced amphibian is the American Bullfrog (*Rana catesbeiana*), and the two introduced lizards are species of the family Gekkonidae: the Stump-toed Gecko (*Gehyra mutilata*) and the Common House Gecko (*Hemidactylus frenatus*). There are also 20 species (eight amphibians and 12 reptiles) that potentially occur within the state of Colima (Table 3). Most of these are species from the northern slope of Nevado de Colima in Jalisco, and it is highly likely that they also occur on the southern slope of this volcano in Colima. Some other species have been recorded in extreme southwestern Jalisco, near the border with Colima and it is likely that they occur in extreme western Colima. There are a few other species that might occur in eastern or southern Colima, near the border with the states of Jalisco and Michoacán. We are confident that with more samples in these areas with low accessibility they will be recorded in Colima, resulting in a much richer herpetological species list. This conclusion of a richer herpetofauna in Colima than currently documented is supported by the species accumulation curves we generated (Fig. 6). The species accumulation curves show a general and continuous increase in the number of species known in Colima during the first half of the 20^th^ century followed by a plateau in the second half of the 20^th^ century; however, there has been a rapid upturn in new species being documented in the 21^st^ century, suggesting the total number of amphibians and reptiles in Colima is likely to be higher, perhaps substantially, than the 153 species we document here. These results make it clear that continued exploration and surveying of the amphibians and reptiles of Colima are needed to establish a firm understanding of their richness in the state.

### General distribution

Twenty-six of the 39 species of amphibians that inhabit Colima are endemic to Mexico (Table 1). One to northern Colima near the state line with Jalisco at the Sierra de Manantlán (*Eleutherodactylus manatlanensis*). Seven are restricted to small areas in northern, eastern, or southern Colima and adjacent Jalisco or Michoacán, or both. Twelve are species typical of the Mexican Pacific, extending from northwestern Mexico (Chihuahua, Sonora or Sinaloa) to the Balsas Depression or even Oaxaca or Chiapas. Four have a wide distribution in northern and central Mexico, and two occur along both coasts of Mexico. Of the 14 species not endemic to Mexico, four are distributed from the US to central or southern Mexico, four are distributed from the US to Central or South America, four are found in Mexico and Central or South America, and one is an introduced species, the American Bullfrog (*Rana catesbeiana*).

The American Crocodile (*Crocodylus acutus*) is widely distributed from the eastern US to South America, including the Caribbean. Two of the 41 species of lizards that occur in Colima are endemic to islands of the Revillagigedo Archipielago (*Urosaurus auriculatus* on Socorro Island and *U. clarionensis* on Clarion Island), and 24 are endemic to Mexico (Table 1). Of the 15 lizard species not endemic to Mexico that inhabit Colima, two are introduced, one is found in the US and Mexico, one is found from the US to Central America, and 11 have a wide distribution that includes Mexico and Central or South America (Table 1). Two of the 64 species of snakes found in Colima are endemic to Isla Clarion in the Revillagigedo Archipielago (*Masticophis anthonyi* and *Hypsiglena unaocularis*) (Table 1). Forty-two of the snake species found in Colima are endemic to Mexico. Of the 22 snake species not endemic to Mexico that occur in Colima, three are distributed from the US to Mexico, four from the US to Central or South America, 12 from Mexico to Central or South America, and one marine species is distributed in the Pacific and Indian Oceans (Table 1). Three of the eight species of turtles found in Colima are endemic to Mexico (Table 1). One is distributed from Mexico to Central America, and four are sea turtles that have a circumglobal distribution (Table 1).

### Conservation status

**Figure 7.**
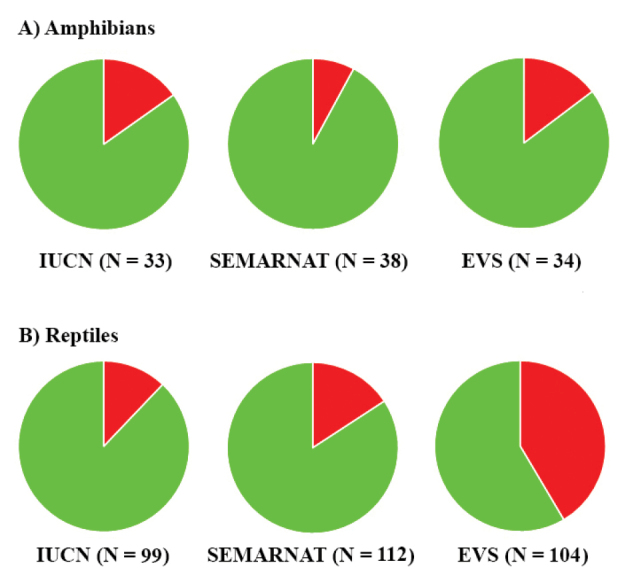
Proportion of **A** amphibians and **B** reptiles listed in protected categories on the IUCN Red List, SEMARNAT, and high EVS. Green is proportion in Data Deficient and Least Concern (IUCN); Not Listed and Subject to Special Protection (we regarded the category of Subject to Special Protection in SEMARNAT equivalent to Least Concern in IUCN) (SEMARNAT); or low or medium EVS. Red is percentage in protected categories or high EVS. N is the number of species assessed.

Of the amphibians and reptiles found in Colima, 12.9% are IUCN listed (i.e., Vulnerable, Near Threatened, Endangered, or Critically Endangered), and 14.0% are placed in a protected category by SEMARNAT (excluding NL and Pr, this last category is equivalent to the LC category of IUCN), and 34.1% are categorized as high risk by the EVS (Tables 1, 2). For amphibians, 15.2% are IUCN listed, 7.9% are protected by SEMARNAT, and 14.7% are at high risk according to the EVS (Fig. 7; Tables 1, 2). For reptiles, 12.1% are listed by the IUCN, 16.1% are protected by SEMARNAT, and 40.4% are at high risk according to the EVS (Fig. 7; Tables 1, 2). These results suggest that the herpetofauna as a whole of Colima is considered to be a relatively low conservation concern at a global scale (i.e., IUCN listing) and national level (i.e., SEMARNAT listing, EVS). However, the EVS categories suggest that, at a national level, the reptiles of Colima are at higher risk than the other assessments suggest and are at higher risk than the amphibians of Colima. In addition, there are several specific taxa that, based on their IUCN listing, SEMARNAT category, or their EVS, are of conservation concern. These include species in the families Eleutherodactylidae, Ranidae, Plethodontidae, Crocodylidae, Eublepharidae, Helodermatidae, Iguanidae, Phrynosomatidae, Phyllodactylidae, Xantusidae, Colubridae, Natricidae, Viperidae, Cheloniidae, and Dermochelyidae (Tables 1, 2). In particular, the family Eleutherodactylidae in Colima is of great conservation concern; this family has seven species, three of them are IUCN listed and are at great risk according to their EVS (*Eleutherodactylus angustidigitorum*, *E. modestus*, and *E. rufescens*). Another three have not been evaluated by the IUCN or EVS due to their recent description (*E. colimotl*, *E. grunwaldi*, and *E. manantlanensis*); however, due to their limited distribution it is almost certain that once they are evaluated, they will be considered in some category of the IUCN and with a high risk EVS, therefore the family Eleutherodactylidae in Colima would be represented by six species (86% = 6/7) at high conservation risk. Because the summarized conservation statuses are global or national-level assessments, the conservation status of at least some species of amphibians and reptiles in Colima are probably not accurately assessed by these measures. Additional assessments at the state level will be required to establish conservation or management needs for the herpetofauna of Colima.

**Table 1.** Amphibians and reptiles of Colima with distributional information and conservation status. Physiographic region: (1 = Volcanes de Colima; 2 = Sierras de la Costa de Jalisco y Colima; 3 = Cordillera Costera del Sur; 4 = Marine; 5 = Islands); IUCN Status: (DD = Data Deficient; LC = Least Concern, V = Vulnerable, NT = Near Threatened; E = Endangered; CE = Critically Endangered; NE = not Evaluated) according to the IUCN Red List (The IUCN Red List of Threatened Species, Version 2019-2 (www.iucnredlist.org; accessed 2 October 2019); conservation status in Mexico according to SEMARNAT (2010): (P = in danger of extinction, A = threatened, Pr = subject to special protection, NL – not listed); Environmental Vulnerability Score: (EVS: low (L) vulnerability species (EVS of 3–9); medium (M) vulnerability species (EVS of 10–13); and high (H) vulnerability species (EVS of 14–20) from Wilson et al. (2013a,b) and Johnson et al. (2015). Global Distribution: 0 = Endemic to Colima; 1 = Endemic to Mexico; 2 = Shared between the US and Mexico; 3 = widely distributed from Mexico to Central or South America; 4 = widely distributed from the US to Central or South America; 5 = circumglobal distribution; 6 = Pacific and Indian Oceans; IN = Introduced to Colima. Date in which the first record appeared; and Source of the record.

	Physiographic region	IUCN status	SEMARNAT	EVS	Global distribution	Year of first record	Source
**Class Amphibia (39)**							
**Order Anura (36)**							
**Family Bufonidae (5)**							
*Anaxyrus compactilis* (Wiegmann, 1833)	1, 2, 3	LC	NL	H (14)	1	1935	FMNH 103444
*Incilius marmoreus* (Wiegmann, 1833)	1, 2, 3	LC	NL	M (11)	1	1905	UAZ 11613
*Incilius mazatlanensis* (Taylor, 1940)	1, 2	LC	NL	M (12)	1	1965	UAZ 33286
*Incilius occidentalis* (Camerano, 1879)	1	LC	NL	M (11)	1	1961	UCM 61121
*Rhinella horribilis* (Wiegmann, 1833)	1, 2, 3,	NE	NL	NE	4	1901	MNHN RA 1901.341
**Family Craugastoridae (2)**							
*Craugastor occidentalis* (Taylor, 1941)	1, 2	DD	NL	M (13)	1	1958	UTEP H-14317
*Craugastor vocalis* (Taylor, 1940)	1	LC	NL	M (13)	1	1970	FSM-UF 66361
**Family Eleutherodactylidae (7**)							
*Eleutherodactylus angustidigitorum* (Taylor, 1940)	1	VU	Pr	H (17)	1	1964	LACM 25450
*Eleutherodactylus colimotl* Grünwald, Reyes- Velasco, Franz-Chávez, Morales-Flores, Ahumada-Carrillo, Jones & Boissinot, 2018	2, 3	NE	NL	NE	1	2015	Grünwald et al. (2018)
*Eleutherodactylus grunwaldi* Reyes-Velasco, Ahumada-Carrillo, Burkhardt, & Devitt, 2015	2	NE	NL	NE	1	2011	Reyes-Velasco et al. (2015)
*Eleutherodactylus manantlanensis* Grünwald, Reyes-Velasco, Franz-Chávez, Morales-Flores, Ahumada-Carrillo, Jones & Boissinot, 2018	2	NE	NL	NE	0	2014	Grünwald et al. (2018)
*Eleutherodactylus modestus* (Taylor, 1942)	1, 2	VU	Pr	H (16)	1	1935	USNM 139729
*Eleutherodactylus nitidus* (Peters, 1870)	2, 3	LC	NL	M (12)	1	1956	UMMZ 114311
*Eleutherodactylus rufescens* (Duellman &Dixon, 1959)	1, 2	CR	Pr	H (17)	1	1963	USNM 161162
**Family Hylidae (10)**							
*Dendropsophus sartori* (Smith, 1951)	2, 3	LC	A	H (14)	1	1960	MVZ 71221
*Dryophytes arenicolor* Cope, 1866	2, 3	LC	NL	L (7)	2	1973	UTEP H-10258
*Dryophytes eximius* (Baird, 1854)	1, 2	LC	NL	M (10)	1	1975	UTEP H-10387
*Exerodonta smaragdina* (Taylor, 1940)	2	LC	Pr	M (12)	1	1953	UMMZ 110873
*Exerodonta sumichrasti* Brocchi, 1879	1	LC	NL	L (9)	1	NA	USNM 57518
*Smilisca baudini* (Duméril & Bibron, 1841)	1, 2, 3	LC	NL	L (3)	4	1902	USNM 57555
*Smilisca fodiens* (Boulenger, 1882)	1	LC	NL	L (8)	2	1919	MCZ A-6683
*Tlalocohyla smithii* (Boulenger, 1902)	1, 2, 3	LC	NL	M (11)	1	1960	MVZ 71216
*Trachycephalus vermiculatus* (Cope, 1877)	1, 2	NE	NL	L (4)	3	1935	UMMZ 80018
*Triprion spatulatus* Günther, 1882	1, 2, 3	LC	NL	M (13)	1	1958	UAZ 12869
**Family Leptodactylidae (2)**							
*Leptodactylus fragilis* (Brocchi, 1877)	1, 2	LC	NL	L (5)	4	1958	UTEP H-14352
*Leptodactylus melanonotus* (Hallowell, 1861)	1, 2, 3	LC	NL	L (6)	3	1902	USNM 57765
**Family Microhylidae (2)**							
*Hypopachus ustus* (Cope, 1866)	1, 2	LC	Pr	L (7)	3	1935	UMMZ 79998
*Hypopachus variolosus* (Cope, 1866)	1, 2	LC	NL	L (4)	3	1935	USNM 118658
**Family Phyllomedusidae (1)**							
*Agalychnis dacnicolor* (Cope, 1864)	1, 2	LC	NL	M (13)	1	1963	FSM-UF 109279
**Family Ranidae (6)**							
*Rana berlandieri* Baird, 1859	2	LC	Pr	L(7)	2	1919	FMNH 1628
*Rana catesbeiana* Shaw, 1802	N/A	N/A	N/A	N/A	IN	2018	https://www.youtube
*Rana forreri* Boulenger, 1883	1, 2	LC	Pr	L (3)	4	1964	CAS 97107
*Rana neovolcanica* Hillis & Frost, 1985	1	NT	A	M (13)	1	2016	Cruz-Saenz et al. (2018)
*Rana pustulosa* Boulenger, 1883	1, 2	LC	Pr	L (3)	1	1700	MCZ A-20941
*Rana zweifeli* Hillis, Frost & Webb, 1984	1	LC	NL	M (11)	1	1982	MNHUK 194432
**Family Scaphiopodidae (1)**							
*Spea multiplicata* (Cope, 1863)	1	LC	NL	L (6)	2	1960	TNHC 19343
**Order Caudata (2)**							
**Family Ambystomatidae (1)**							
*Ambystoma velasci* Dugès, 1888	1, 2	LC	Pr	M (10)	1	1868	MNHN RA 1868.175
**Family Plethodontidae (1)**							
*Isthmura belli* (Gray, 1850)	2	VU	A	M (12)	1	1970	MCZ A-85395
**Order Gymnophiona (1)**							
**Family Caecilidae (1)**							
*Dermophis oaxacae* (Mertens, 1930)	1, 2	DD	Pr	M (12)	1	1970	FSM-UF 61604
**Class Reptilia (112)**							
**Order Crocodylia (1)**							
**Family Crocodylidae (1)**							
*Crocodylus acutus* (Cuvier, 1807)	1, 2	VU	Pr	H (14)	4	1892	USNM 52336
**Order Squamata (107)**							
**Suborder Lacertilia (41)**							
**Family Anguidae (3)**							
*Barisia imbricata* (Wiegmann, 1828)	2	LC	Pr	H (14)	1	1965	UAZ 32849
*Elgaria kingii* Gray, 1838	2	LC	Pr	M (10)	2	1993	MZFC 6811
*Gerrhonotus liocephalus* Wiegmann, 1828	1, 2	LC	Pr	L (6)	1	1868	MNHN RA 1868.153
**Family Corytophanidae (2)**							
*Basiliscus vittatus* Wiegmann, 1828	2	LC	NL	L (7)	3	1935	UMMZ 80147
*Laemanctus longipes* Wiegmann, 1834	2	LC	Pr	L (9)	3	1904	FMNH 1353
**Family Dactyloidae (1)**							
*Anolis nebulosus* (Wiegmann, 1834)	1, 2, 3	LC	NL	M (13)	1	1863	USNM 63700
**Family Eublepharidae (1)**							
*Coleonyx elegans* Gray, 1845	2, 3	LC	A	L (9)	3	1960	MNHUK 62400
**Family Gekkonidae (2)**							
*Gehyra mutilata* (Wiegmann, 1834)	1, 2, 3	N/A	N/A	N/A	IN	1976	AMNH R-163687
*Hemidactylus frenatus* Duméril & Bribon, 1836	2	N/A	N/A	N/A	IN	1960	MVZ 71229
**Family Helodermatidae (1)**							
*Heloderma horridum* (Wiegmann, 1829)	1, 2, 3	LC	A	M (11)	3	1818	MVZ 79417
**Family Iguanidae (2)**							
*Ctenosaura pectinata* (Wiegmann, 1834)	1, 2, 3	NE	NL	H (15)	1	1863	MCZ R-24902
*Iguana iguana* (Linnaeus, 1758)	1, 2, 3	LC	Pr	M (12)	3	1863	USNM 63699
**Family Phrynosomatidae (14)**							
*Phrynosoma asio* Cope, 1864	1, 2	LC	Pr	M (11)	1	1935	UMMZ 80067
*Phrynosoma orbiculare* (Linnaeus, 1758)	1	LC	A	M (12)	1	1870	Duméril and Bocourt (1870)
*Sceloporus bulleri* Boulenger, 1895	2	LC	NL	H (15)	1	2004	Reyes-Velasco et al. (2009)
*Sceloporus dugesii* Bocourt, 1874	1, 2	LC	NL	M (13)	1	1969	ASNHC 13801
*Sceloporus horridus* Wiegmann, 1834	1, 2, 3	LC	NL	M (12)	1	1863	USNM 31389
*Sceloporus melanorhinus* Bocourt, 1876	1, 2	LC	NL	L (9)	3	1863	USNM 31496
*Sceloporus nelsoni* Cochran, 1923	2	LC	NL	M (13)	1	1964	LACM 74288
*Sceloporus pyrocephalus* Cope, 1864	1, 2, 3	LC	NL	M (12)	1	1863	USNM 31449
*Sceloporus torquatus* Wiegmann, 1828	2	LC	NL	M (11)	1	1863	MNHN RA 0.2922
*Sceloporus utiformis* Cope, 1864	1, 2, 3	LC	NL	H (15)	1	1902	USNM 58811
*Sceloporus unicanthalis* Smith, 1937	2	NE	NL	H (16)	1	2005	Reyes-Velasco et al. (2009)
*Urosaurus auriculatus* (Cope, 1871)	5	EN	NL	H (16)	0	1871	Cope (1871)
*Urosaurus bicarinatus* (Duméril, 1856)	1, 2, 3	LC	NL	M (12)	1	1919	CAS 54904
*Urosaurus clarionensis* (Townsend, 1890)	5	VU	NL	H (17)	0	1890	Townsend (1890)
**Family Phyllodactylidae (3)**							
*Phyllodactylus davisi* Dixon, 1964	1, 2	LC	A	H (16)	1	1930	MVZ 12186
*Phyllodactylus lanei* Smith, 1935	1, 2	LC	NL	H (15)	1	1937	Oliver (1937)
*Phyllodactylus tuberculosus* Wiegmann, 1834	2	LC	NL	L (8)	3	NA	MNHN RA 0.1657
**Family Scincidae (5)**							
*Marisora brachypoda* (Taylor, 1956)	1, 2, 3	LC	NL	L (6)	3	1962	LACM 5987
*Plestiodon indubitus* (Taylor, 1933)	2	NE	NL	H (15)	1	1923	MCZ R-135422
*Plestiodon colimensis* (Taylor, 1935)	1	DD	Pr	H (14)	1	1935	Taylor (1936)
*Plestiodon parvulus* (Taylor, 1933)	2	DD	NL	H (15)	1	1935	UMMZ 80108
*Scincella assata* (Cope, 1864)	1, 2	LC	NL	L (7)	3	1935	UMMZ 80106
**Family Teiidae (6)**							
*Aspidoscelis communis* (Cope, 1878)	1, 2, 3	LC	Pr	H (14)	1	1920	LACM 7956
*Aspidoscelis costatus* (Cope, 1878)	1, 2	LC	Pr	M (11)	1	1863	USNM 31610
*Aspidoscelis deppii* (Wiegmann, 1834)	1, 2	LC	NL	L (8)	3	1959	UAZ 06297
*Aspidoscelis guttatus* (Wiegmann, 1834)	2	LC	NL	M (12)	1	1969	ASNHC 13965
*Aspidoscelis lineattissimus* (Cope, 1878)	1, 2, 3	LC	Pr	H (14)	1	1957	UCM 14659
*Holcosus sinister* (Wiegmann, 1834)	1, 2, 3	NE	NL	M (13)	1	1920	LACM 7956
**Family Xantusidae (1)**							
*Lepidophyma tarascae* Bezy, Webb & Álvarez, 1982	3	DD	A	H (14)	1	2005	Reyes-Velasco et al. (2009)
**Suborder Serpentes (66)**							
**Family Boidae (1)**							
*Boa sigma* Smith, 1943	1, 2	NE	NL	H (15)	1	1863	USNM 62024
**Family Colubridae (23)**							
*Conopsis biserialis* (Taylor & Smith, 1942)	2	LC	A	M (13)	1	2004	Reyes-Velasco et al. (2009)
*Drymarchon melanurus* (Duméril, Bibron & Duméril, 1854)	1, 2	LC	NL	L (6)	3	1902	CM S7254
*Drymobius margaritiferus* (Schlegel, 1837)	1, 2	LC	NL	L (6)	3	1902	CM S7252
*Geagras redimitus* Cope, 1875	2	DD	Pr	H (14)	1	1962	MVZ 75805
*Lampropeltis polyzona* Cope, 1860	1	LC	NL	L(7)	1	1863	MCZ R-27105
*Leptophis diplotropis* (Günther, 1872)	2	LC	A	H (14)	1	1962	MVZ 75804
*Masticophis anthonyi* (Stejneger, 1901)	5	CR	A	H (17)	0	1901	Stejneger (1901)
*Masticophis bilineatus* (Jan, 1863)	1, 2	LC	NL	M (11)	2	1914	MCZ R-11409
*Masticophis mentovarius* (Duméril, Bibron & Duméril, 1854)	1, 2	LC	A	L (6)	3	1863	USNM 32234
*Mastigodryas melanolomus* (Cope, 1868)	1, 2	LC	NL	L (6)	3	1902	USNM 56283
*Oxybelis aeneus* (Wagler, 1824)	2	LC	NL	L (5)	4	1892	USNM 46606
*Pituophis deppei* (Dumeril, 1853)	1	LC	A	H (14)	1	1868	MNHN RA 1868.157
*Pseudoficimia frontalis* (Cope, 1864)	1, 2, 3	LC	NL	M (13)	1	1956	UMMZ 114482
*Salvadora lemniscata* (Cope, 1895)	2	LC	Pr	H (15)	1	1971	CAS 132121
*Salvadora mexicana* (Duméril, Bibron & Duméril, 1854)	1, 2, 3	LC	Pr	H (15)	1	1863	USNM 61969
*Senticolis triaspis* (Cope, 1866)	1, 2	LC	NL	L (6)	4	1935	UMMZ 80210
*Sonora michoacanensi* (Dugès, 1884)	2	LC	NL	H (14)	1	1966	Harris and Simmons (1970)
*Symphimus leucostomus* Cope, 1869	3	LC	Pr	H (14)	1	2004	Reyes-Velasco et al. (2009)
*Tantilla bocourti* (Günther, 1895)	3	LC	NL	L (9)	1	1960	MVZ 72202
*Tantilla calamarina* Cope, 1866	1, 2	LC	Pr	M (12)	1	1935	UMMZ 80224
*Tantilla ceboruca* Canseco-Marquéz, Smith, Ponce-Campos, Flores-Villela & Campbell, 2007	1	NE	NL	H (16)	1	2004	Reyes-Velasco et al. (2012)
*Trimorphodon biscutatus* (Duméril, Bibron & Duméril, 1854)	1, 2, 3	NE	NL	L (7)	3	1818	MVZ 72194
*Trimorphodon tau* Cope, 870	1, 2	LC	NL	M (13)	1	1956	UMMZ 114479
**Family Dipsadidae (21)**							
*Clelia scytalina* (Cope, 1867)	1, 2	LC	NL	M (13)	3	1963	MVZ 76355
*Coniophanes lateritius* Cope, 1862	2	DD	NL	M (13)	1	2005	Reyes-Velasco et al. (2009)
*Conophis vittatus* Peters, 1860	1, 2	LC	NL	M (11)	1	1961	FSM-UF 42088
*Dipsas gaigeae* (Oliver, 1937)	2	LC	Pr	H (17)	1	1935	UMMZ 80221
*Enulius flavitorques* (Cope, 1868)	2	LC	NL	L (5)	3	1959	UAZ 20369
*Geophis dugesii* Boucourt, 1883	1	LC	NL	M (13)	1	1914	MCZ R-11422
*Geophis sieboldi* (Jan, 1862)	1	DD	Pr	M (13)	1	2012	Ahumada-Carrillo et al. (2014)
*Hypsiglena torquata* (Günther, 1860)	1, 2	LC	Pr	L (8)	1	1968	MNHN RA 1868.162
*Hypsiglena unaocularus* Tanner, 1946	5	NE	NL	NE	0	1946	Tanner (1946)
*Imantodes gemmistratus* (Cope, 1861)	1, 2	LC	Pr	L (6)	3	1935	UMMZ 80215
*Leptodeira maculata* (Hallowell, 1861)	1, 2	LC	Pr	L (7)	1	1863	USNM 31486
*Leptodeira septentrionalis* (Kennicott, 1859)	2	LC	NL	L (8)	4	1935	UMMZ Herps 80220
*Leptodeira splendida* Günther, 1895	1, 2	LC	NL	H (14)	1	1914	MCZ R-11411
*Leptodeira uribei* (Ramírez-Bautista & Smith, 1992)	2	LC	NL	H (17)	1	2004	Reyes-Velasco et al. (2009)
*Manolepis putnami* (Jan, 1863)	2	LC	NL	M (13)	1	1863	USNM 31478
*Pseudoleptodeira latifasciata* (Günther, 1894)	3	LC	Pr	H (14)	1	1961	MNHUK 63423
*Rhadinaea hesperia* Bailey, 1940	2	LC	Pr	M (10)	1	1935	UMMZ 80226
*Rhadinaea taeniata* (Peters, 1863)	1, 2	LC	NL	M (13)	1	1969	CAS 121078
*Sibon nebulatus* (Linnaeus, 1758)	1, 3	NE	NL	L (5)	3	1960	USNM 196500
*Tropidodipsas annulifera* Boulenger, 1894	1, 2, 3	LC	Pr	M (13)	1	2004	Reyes-Velasco et al. (2009)
*Tropidodipsas philippii* (Jan, 1863)	1, 2	LC	Pr	H (14)	1	1914	MCZ R-11410
**Family Elapidae (6)**							
*Hydrophis platurus* (Linnaeus, 1766)	4	LC	NL	NE	6	1956	UMMZ 114561
*Micrurus browni* Schmidt & Smith, 1943	2	LC	Pr	L (8)	3	1976	NLU 40764
*Micrurus distans* Kennicott, 1860	1, 2	LC	Pr	H (14)	1	1914	MCZ R-11416
*Micrurus laticollaris* Peters, 1870	1, 2, 3	LC	Pr	H (14)	1	1951	MNHUK 32546
*Micrurus proximans* Smith & Chrapliwy, 1958	2	LC	Pr	H (18)	1	2008	Reyes-Velasco et al. (2012)
*Micrurus tener* Baird & Girard, 1953	1	LC	NL	M (11)	2	2004	Reyes-Velasco et al. (2009)
**Family Leptotyphlopidae (2)**							
*Epictia bakewelli* (Oliver, 1937)	1, 2	NE	NL	NE	1	1935	UMMZ 80228
*Rena dugesii* (Bocourt, 1881)	2	NE	NL	NE	2	1868	MNHN RA 1868.154
**Family Loxocemidae (1)**							
*Loxocemus bicolor* Cope, 1861	1, 2	LC	Pr	M (10)	3	1863	USNM 61924
**Family Natricidae (4)**							
*Storeria storerioides* (Cope, 1866)	2	LC	NL	M (11)	1	2004	Reyes-Velasco et al. (2009)
*Thamnophis cyrtopsis* (Kennicott, 1860)	1	LC	A	L (7)	4	1964	LSUMZ 7846
*Thamnophis melanogaster* (Wiegmann, 1830)	1	EN	A	H (15)	1	1868	MNHN RA 1868.161
*Thamnophis validus* (Kennicott, 1860)	2	NE	NL	M (12)	1	1961	MNHUK 63428
**Family Viperidae (6)**							
*Agkistrodon bilineatus* Günther, 1863	1, 2	NT	Pr	M (11)	3	1928	UMMZ 68433
*Crotalus basiliscus* (Cope, 1864)	1, 2	LC	Pr	H (16)	1	1864	Cope (1864)
*Crotalus campbelli* Bryson, Linkem, Dorcas, Lathrop, Jones, Alvarado-Díaz, Grünwald & Murphy, 2014	2	NE	NL	H (17)	1	2004	Reyes-Velasco et al. (2009)
*Crotalus lannomi* Tanner, 1966	2	DD	A	H (19)	1	2008	Reyes-Velasco et al. (2010)
*Crotalus pusillus* Klauber, 1952	1	EN	A	H (18)	1	2008	Reyes-Velasco et al. (2012)
*Porthidium hespere* (Campbell, 1976)	2, 3	DD	Pr	H (18)	1	1973	Campbell (1976)
**Order Testudines (8)**							
**Family Cheloniidae (3)**							
*Caretta caretta* (Linnaeus, 1758)	4	VU	P	NE	5	NA	UMMZ40350
*Chelonia mydas* (Linnaeus, 1758)	4	EN	P	NE	5	1905	CAS 8532
*Lepidochelys olivacea* (Eschscholtz, 1829)	4	VU	P	NE	5	1964	LACM 8111
**Family Dermochelyidae (1)**							
*Dermochelys coriacea* (Vandelli, 1761)	4	VU	P	NE	5	1971	AMNH R-172553
**Family Geoemydidae (2)**							
*Rhinoclemmys pulcherrima* (Gray, 1855)	1, 2	NE	NL	L (8)	3	1935	UMMZ 80348
*Rhinoclemmys rubida* (Cope, 1870)	1, 2	NT	Pr	H (14)	1	1902	CAS 14085
**Family Kinosternidae (2)**							
*Kinosternon chimalhuaca* Berry, Seidel &Iverson, 1997	2	LC	NL	H (16)	1	1997	Berry et al. (1997)
*Kinosternon integrum* LeConte, 1854	1, 2	LC	Pr	M (11)	1	1892	USNM 50990

**Table 2.** Summary of native species present in Colima by Family, Order or Suborder, and Class. Status summary indicates the number of species found in each IUCN conservation status in the Order DD, LC, VU, NT, EN, CE (see Table 1 for abbreviations; in some cases species have not been assigned a status by the IUCN and therefore these may not add up to the total number of species in a taxon). Mean EVS is the mean Environmental Vulnerability Score, scores ≥ 14 are considered high vulnerability (Wilson et al. 2013a, b) and conservation status in Mexico according to SEMARNAT (2010) in the Order NL, Pr, A, P (see Table 1 for abbreviations).

Scientific name	Genera	Species	IUCN	mean EVS	SEMARNAT
**Class Amphibia**					
**Order Anura**	**17**	**35**	**1, 25, 2, 1, 0, 1**	**9.8**	**25, 8, 2, 0**
Bufonidae	3	5	0, 4, 0, 0, 0, 0	12	5, 0, 0, 0
Craugastoridae	1	2	1, 1, 0, 0, 0, 0	13	2, 0, 0, 0
Eleutherodactylidae	1	7	0, 1, 2, 0, 0, 1	15.5	4, 3, 0, 0
Hylidae	7	10	0, 9, 0, 0, 0, 0	9.1	8, 1, 1, 0
Leptodactylidae	1	2	0, 2, 0, 0, 0, 0	5.5	2, 0, 0, 0
Microhylidae	1	2	0, 2, 0, 0, 0, 0	5.5	1, 1, 0, 0
Phyllomedusidae	1	1	0, 1, 0, 0, 0, 0	13	1, 0, 0, 0
Ranidae	1	5	0, 4, 0, 1, 0, 0	7.4	1, 3, 1, 0
Scaphiopodidae	1	1	0, 1, 0, 0, 0, 0	6	1, 0, 0, 0
**Order Caudata**	**2**	**2**	**0, 1, 1, 0, 0, 0**	**11**	**0, 1, 1, 0**
Ambystomatidae	1	1	0, 1, 0, 0, 0, 0	10	0, 1, 0, 0
Plethodontidae	1	1	0, 0, 1, 0, 0, 0	12	0, 0, 1, 0
**Order Gymnophiona**	**1**	**1**	**1, 0, 0, 0, 0, 0**	12	**0, 1, 0, 0**
Caecilidae	1	1	1, 0, 0, 0, 0, 0	12	0, 1, 0, 0
**Subtotal**	**20**	**38**	**2, 26, 3, 1, 0, 1**	**10.0**	**25, 10, 3, 0**
**Class Reptilia**					
**Order Crocodylia**	**1**	**1**	**0, 0, 1, 0, 0, 0**	**14**	**0, 1, 0, 0**
Crocodylidae	1	1	0, 0, 1, 0, 0, 0	14	0, 1, 0, 0
**Order Squamata**	**62**	**103**	**8, 77, 1, 1, 3, 1**	**12**	**57, 32, 14, 0**
**Suborder Lacertilia**	**20**	**39**	**3, 30, 1, 0, 1, 0**	**12.1**	**24, 10, 5, 0**
Anguidae	3	3	0, 3, 0, 0, 0, 0	10	0, 3, 0, 0
Corytophanidae	2	2	0, 2, 0, 0, 0, 0	8	1, 1, 0, 0
Dactyloidae	1	1	0, 1, 0, 0, 0, 0	13	1, 0, 0, 0
Eublepharidae	1	1	0, 1, 0, 0, 0, 0	9	0, 0, 1, 0
Helodermatidae	1	1	0, 1, 0, 0, 0, 0	11	0, 0, 1, 0
Iguanidae	2	2	0, 1, 0, 0, 0, 0	13.5	1, 1, 0, 0
Phrynosomatidae	3	14	0, 11, 1, 0, 1, 0	13.1	12, 1, 1, 0
Phyllodactylidae	1	3	0, 3, 0, 0, 0, 0	13	2, 0, 1, 0
Scincidae	3	5	2, 2, 0, 0, 0, 0	11.4	4, 1, 0, 0
Teiidae	2	6	0, 5, 0, 0, 0, 0	12	3, 3, 0, 0
Xantusidae	1	1	1, 0, 0, 0, 0, 0	14	0, 0, 1, 0
**Suborder Serpentes**	**42**	**64**	**5, 47, 0, 1, 2, 1**	**11.9**	**33, 22, 9, 0**
Boidae	1	1	0, 0, 0, 0, 0, 0	15	1, 0, 0, 0
Colubridae	17	23	1, 19, 0, 0, 0, 1	11	13, 5, 5, 0
Dipsadidae	14	21	2, 18, 0, 0, 0, 0	11.4	12, 9, 0, 0
Elapidae	2	6	0, 6, 0, 0, 0, 0	13	2, 4, 0, 0
Leptotyphlopidae	2	2	0, 0, 0, 0, 0, 0	NE	2, 0, 0, 0
Loxocemidae	1	1	0, 1, 0, 0, 0, 0	10	0, 1, 0, 0
Natricidae	2	4	0, 2, 0, 0, 1, 0	11.3	2, 0, 2, 0
Viperidae	3	6	2, 1, 0, 1, 1, 0	16.5	1, 3, 2, 0
**Order Testudines**	**6**	**8**	**0, 2, 3, 1, 1, 0**	**12.3**	**2, 2, 0, 4**
Cheloniidae	3	3	0, 0, 2, 0, 1, 0	NE	0, 0, 0, 3
Dermochelyidae	1	1	0, 0, 1, 0, 0, 0	NE	0, 0, 0, 1
Geoemydidae	1	2	0, 0, 0, 1, 0, 0	11	1, 1, 0, 0
Kinosternidae	1	2	0, 2, 0, 0, 0, 0	13.5	1, 1, 0, 0
**Subtotal**	**69**	**112**	**8, 79, 5, 2, 4, 1**	**12**	**59, 35, 14, 4**
**Total**	**89**	**150**	**10, 105, 8, 3, 4, 2**	**11.5**	**84, 45, 17, 4**

Using the data in Table 1, we summarized the conservation status of amphibian and reptile taxa in each physiographic region found in Colima. For IUCN listing, 13.8% of the amphibians in the Volcanes de Colima physiographic region are listed; 10.0% in the Sierras de la Costa de Jalisco y Colima; and none in the Cordillera Costera del Sur. For SEMARNAT categories, 3.4% of amphibian species in the Volcanes de Colima are listed; 6.7% in the Sierras de la Costa de Jalisco y Colima; and 9.1% in the Cordillera Costera del Sur. For EVS, 13.8% of the amphibians in the Volcanes de Colima in the high-risk category; 13.3% in the Sierras de la Costa de Jalisco y Colima, and 18.2% in the Cordillera Costera del Sur. For IUCN listings, relatively few species of reptiles are placed in the protected categories for most of the physiographic regions (Volcanes de Colima, 7.8%; Sierras de la Costa de Jalisco y Colima, 3.4%; Cordillera Costera del Sur, 0%). Reptiles in the Marine (80%) and Revillagigedo Archipelago (75%) regions show relatively high percentages of species in protected categories. Similar patterns hold for SEMARNAT listings with 12.5% of reptiles in the Volcanes de Colima, 8.0% from Sierras de la Costa de Jalisco y Colima, and 12.5% from the Cordillera Costera del Sur, 80% in the Marine region, and 25% in the Revillagigedo Archipelago region in the protected SEMARNAT categories. For the EVS assesments of reptile species, 31.3% were in the high category in Volcanes de Colima, 35.6% in the Sierras de la Costa de Jalisco y Colima, 41.7% in the Cordillera Costera del Sur. None of the five species in the marine region were evaluated for EVS, and 75% of the species in the Revillagigedo Archipelago were in the high EVS category. Based on our summary of conservation status, the reptiles in the Marine and Revillagigedo Archipelago regions are the most threatened taxa of the Colima herpetofauna.

**Table 3.** List of amphibians and reptiles that potentially occur in Colima.

Taxon	Explanation
**Class Amphibia**	
**Order Anura**	
** Bufonidae **	
*Incilius perplexus* (Taylor, 1943)	Likely to occur in eastern Colima
** Craugastoridae **	
*Craugastor augusti* (Dugès, 1879)	Likely to occur in extreme western Colima
*Craugastor hobartsmithi* (Taylor, 1937)	Likely to occur in extreme western Colima
*Craugastor pygmaeus* (Taylor, 1937)	Likely to occur in northwestern and southern Colima
** Hylidae **	
*Sarcohyla bistincta* (Cope, 1877)	Likely to occur through the state but the coastal area
** Ranidae **	
*Rana megapoda* Taylor, 1942	Likely to occur in northeastern Colima, in the Volcanes de Colima physiographic region
*Rana psilonota* Webb, 2001	Likely to occur in northeastern Colima, in the Volcanes de Colima physiographic region
**Order Caudata**	
** Plethodontidae **	
*Pseudoeurycea leprosa* (Cope, 1869)	Likely to occur in southern Colima
**Class Reptilia**	
**Order Squamata**	
**Suborder Lacertilia**	
** Phrynosomatidae **	
*Sceloporus grammicus* Wiegmann, 1828	Likely to occur in northeastern Colima, in the Volcanes de Colima physiographic region
*Sceloporus heterolepis* Boulenger, 1895	Likely to occur in northeastern Colima, in the Volcanes de Colima physiographic region
**Order Squamata**	
**Suborder Serpentes**	
** Colubridae **	
*Salvadora bairdi* Jan, 1860	Likely to occur in northeastern Colima, in the Volcanes de Colima physiographic region
*Sonora mutabilis* Stickel, 1943	Likely to occur in northeastern-eastern Colima
** Dipsadidae **	
*Geophis bicolor* Günther, 1868	Likely to occur in northeastern Colima, in the Volcanes de Colima physiographic region
*Geophis nigrocinctus* Duellman, 1959	Likely to occur in northern Colima
*Geophis petersi* Boulenger, 1894	Likely to occur in northern Colima
*Geophis tarascae* Hartweg, 1959	Likely to occur in northeastern Colima, in the Volcanes de Colima physiographic region
** Viperidae **	
*Crotalus armstrongi* (Campbell, 1979)	Likely to occur in northeastern Colima, in the Volcanes de Colima physiographic region
*Crotalus culminatus* Klauber, 1952	Likely to occur in southern Colima, near the border with Coahuayana, Michoacán
*Crotalus polystictus* (Cope, 1865)	Likely to occur in northeastern Colima, in the Volcanes de Colima physiographic region
**Order Testudines**	
** Cheloniidae **	
*Eretmochelys imbricata* (Linnaeus, 1766)	Likely to occur in the coastline of the state

### Comparison with neighboring states

For amphibians, Colima shares 92.1% of its species with Jalisco, and it shares 86.8% of its species with Michoacán (Table 4). Species in eight of the 12 families of amphibians present in Colima are fully shared with Jalisco and Michoacán. The percentage of shared reptile species is slightly smaller; however, overlap in species lists is still very high. Colima shares 84.8% of its reptile species with Jalisco, and 82.1% with Michoacán. Species in 13 of the 25 families of reptiles present in Colima are fully shared with Jalisco and Michoacán. Only ten of the species found in Colima (two amphibians and eight reptiles) do not occur in either Jalisco or Michoacán, four of which are species endemic to the Revillagigedo Archipelago (*Urosaurus auriculatus*, *U. clarionensis*, *Masticophis anthonyi*, and *Hypsiglena unaocularus*), one is endemic to northern Colima (*Eleutherodactylus manantlanensis*), three have spotty distributions along the Pacific Coast of Mexico (*Phyllodactylus tuberculosus*) or in south-southeastern Mexico (*Laemanctus longipes* and *Aspidoscelis guttatus*), and two have isolated records in Colima, with the bulk of their distribution in southeastern Mexico (*Exerodonta sumichrasti* and *Salvadora lemniscata*). The high level of similarity in the herpetofauna between Colima and its two neighbors is due in part to the small size of Colima compared with each one of these two state (7.2% of Jalisco, 9.6% of the Michoacán). In addition, and perhaps more importantly, Colima is completely surrounded by Jalisco and Michoacán, and shares the same physiographic regions and habitat types with them.

**Table 4.** Summary of the numbers of species shared between Colima and neighboring Mexican states (not including introduced species). The percent of Colima species shared by a neighboring state are given in parentheses. Total refers to the total number of species found in Colima and two neighboring states (i.e., regional species pool) and the number in parentheses in this column is the percent of the regional species pool found in Colima. – indicates either Colima or the neighboring state has no species in the taxonomic group, thus no value for shared species is provided.

Taxon	Colima	Jalisco	Michoacán	Total
Class Amphibia	38	35 (92.1)	33 (86.8)	73 (52.1)
Order Anura	35	32 (91.4)	30 (85.7)	61 (57.4)
Bufonidae	5	5 (100)	4 (80)	10 (50)
Craugastoridae	2	2 (100)	2 (100)	5 (40)
Eleutherodactylidae	7	6 (85.7)	5 (71.4)	15 (46.6)
Hylidae	10	9 (90.0)	8 (80.0)	13 (76.9)
Leptodactylidae	2	2 (100)	2 (100)	2 (100)
Microhylidae	2	2 (100)	2 (100)	2 (100)
Phyllomedusidae	1	1 (100)	1 (100)	1 (100)
Ranidae	5	4 (80)	5 (100)	11 (45.5)
Rhinophrynidae	0	–	0 (0)	1 (0)
Scaphiopodidae	1	1 (100)	1 (100)	1 (100)
Order Caudata	2	2 (100)	2 (100)	11 (18.2)
Ambystomatidae	1	1 (100)	1 (100)	8 (12.5)
Plethodontidae	1	1 (100)	1 (100)	3 (33.3)
Order Gymnophiona	1	1 (100)	1 (100)	1 (100)
Caecilidae	1	1 (100)	1 (100)	1 (100)
Class Reptilia	112	95 (84.8)	92 (82.1)	211 (53.1)
Order Crocodylia	1	1 (100)	1 (100)	1 (100)
Crocodylidae	1	1 (100)	1 (100)	1 (100)
Order Squamata	103	86 (83.5)	85 (82.5)	198 (52)
Suborder Lacertilia	39	30 (76.9)	30 (76.9)	77 (50.6)
Anguidae	3	3 (100)	3 (100)	7 (42.9)
Corytophanidae	2	1 (50)	1 (50)	2 (100)
Dactyloidae	1	1 (100)	1 (100)	2 (50)
Eublepharidae	1	1 (100)	1 (100)	1 (100)
Helodermatidae	1	1 (100)	1 (100)	1 (100)
Iguanidae	2	2 (100)	2 (100)	3 (66.7)
Phrynosomatidae	14	11 (78.6)	9 (64.3)	34 (41.2)
Phyllodactylidae	3	1 (33.3)	2 (66.7)	6 (50)
Scincidae	5	4 (80)	4 (80)	10 (50)
Teiidae	6	5 (83.6)	5 (83.6)	9 (66.7)
Xantusidae	1	0 (0)	1 (100)	2 (50)
Suborder Serpentes	64	56 (87.5)	55 (85.9)	121 (52.9)
Boidae	1	1 (100)	1 (100)	1 (100)
Colubridae	23	19 (82.6)	19 (82.6)	39 (59)
Dipsadidae	21	20 (95.2)	19 (90.5)	41 (51.2)
Elapidae	6	5 (83.6)	4 (66.7)	7 (85.7)
Leptotyphlopidae	2	1 (50)	2 (100)	4 (50)
Loxocemidae	1	1 (100)	1 (100)	1 (100)
Natricidae	4	4 (100)	4 (100)	12 (33.3)
Viperidae	6	5 (83.3)	5 (83.3)	16 (37.5)
Order Testudines	8	8 (100)	6 (75)	12 (66.7)
Cheloniidae	3	3 (100)	2 (66.7)	4 (75)
Dermochelyidae	1	1 (100)	1 (100)	1 (100)
Emydidae	0	0 (0)	–	2 (0)
Geoemydidae	2	2 (100)	2 (100)	2 (100)
Kinosternidae	2	2 (100)	1 (50)	3 (66.7)
**Total**	**150**	**130 (86.7)**	**125 (83.3)**	**284 (52.8)**

## Conclusions

Colima is home to a rich herpetofauna, especially relative to its small size, and is likely richer than currently known. Its herpetofauna contains a relatively high number of species that are endemic to Mexico, and thus is an important state for the Mexican herpetofauna. Based on IUCN and SEMARNAT listings, the conservation status of the amphibians and reptiles would appear to be relatively low, but the EVS assessments suggest this may not be a completely accurate impression. Of particular concern are the marine species and those species found in the Revillagigedo Archipelago. Colima shares the vast majority of its species with the neighboring states of Jalisco and Michoacán, suggesting that these three states may make a useful unit for understanding and creating conservation and management plans and strategies for their amphibians and reptiles.

## Appendix 1

Museum collections included in the CONABIO database examined for records of Colima amphibians and reptiles or that house specimens of the first record of a species in Colima.

**AMNH** Collection of Herpetology, Herpetology Department, American Museum of Natural History

**ASNHC** Amphibians and Reptiles Collection, Angelo State Natural History Collection

**CAS** Collection of Herpetology, Herpetology Department, California Academy of Sciences

**CMNH** Collection of Herpetology, Amphibian and Reptile Section, Carnegie Museum of Natural History, Pittsburgh

**FMNH** Division of Amphibians and Reptiles, Field Museum of Natural History

**FSM-UF** Collection of Herpetology, Florida State Museum, University of Florida

**LACM** Collection of Herpetology, Herpetology Section, Natural History Museum of Los Angeles County

**LSUMZ** Collection of Herpetology, Museum of Zoology, Biological Science Division, Louisiana State University

**MCZ** Collection of Herpetology, Museum of Comparative Zoology, Harvard University Cambridge

**MNHN** Collection of Reptiles and Amphibians, Muséum National D’Histoire Naturelle

**MNHUK** Museum of Natural History, Division of Herpetology, University of Kansas

**MVZ** Collection of Herpetology, Museum of Vertebrate Zoology, Division of Biological Sciences, University of California Berkeley

**MZFC-UNAM** Colección Herpetológica, Museo de Zoología “Alfonso L. Herrera”, Facultad de Ciencias UNAM

**NLU** Northeastern Louisiana University

**TNHC** Collection of Herpetology, Texas Natural History Collection, University of Texas Austin

**UAZ** Amphibians and Reptiles Collections, University of Arizona

**UCM** Collection of Herpetology, University of Colorado Museum

**UMMZ** Collection of Herpetology, Museum of Zoology, University of Michigan Ann Arbor

**USNM** Collection of Herpetology, Department of Vertebrate Zoology, National Museum of Natural History, Smithsonian Institution

**UTEP** Collection of Herpetology, Laboratory of Environmental Biology, Biological Science Department, University of Texas – El Paso
